# The Hygiene Hypothesis – Learning From but Not Living in the Past

**DOI:** 10.3389/fimmu.2021.635935

**Published:** 2021-03-16

**Authors:** Petra I. Pfefferle, Corinna U. Keber, Robert M. Cohen, Holger Garn

**Affiliations:** ^1^Comprehensive Biobank Marburg, Medical Faculty, Philipps University of Marburg, Comprehensive Biobank Marburg, Marburg, Germany; ^2^German Center for Lung Research (DZL), Marburg, Germany; ^3^German Biobank Alliance, Marburg, Germany; ^4^Institute for Pathology, Medical Faculty, Institute for Pathology, Philipps University of Marburg, Marburg, Germany; ^5^Translational Inflammation Research Division & Core Facility for Single Cell Multiomics, Medical Faculty, Biochemical Pharmacological Center, Philipps University of Marburg, Marburg, Germany

**Keywords:** hygiene hypothesis, allergy, asthma, immune tolerance, T cell-response, microbiome

## Abstract

Postulated by Strachan more than 30 years ago, the Hygiene Hypothesis has undergone many revisions and adaptations. This review journeys back to the beginnings of the Hygiene Hypothesis and describes the most important landmarks in its development considering the many aspects that have refined and generalized the Hygiene Hypothesis over time. From an epidemiological perspective, the Hygiene Hypothesis advanced to a comprehensive concept expanding beyond the initial focus on allergies. The Hygiene Hypothesis comprise immunological, microbiological and evolutionary aspects. Thus, the original postulate developed into a holistic model that explains the impact of post-modern life-style on humans, who initially evolved in close proximity to a more natural environment. Focusing on diet and the microbiome as the most prominent exogenous influences we describe these discrepancies and the resulting health outcomes and point to potential solutions to reestablish the immunological homeostasis that frequently have been lost in people living in developed societies.

Last year we celebrated the 30th anniversary of the Hygiene Hypothesis. Since Strachan framed the Hygiene Hypothesis in 1989 (1) his fundamental idea to explain the origins of allergic diseases development has survived the test of time. The basic idea of how humans, their microbiota, and a continuously modernizing environment have interacted to drive immune dysregulation has persisted and become part of the popular imagination. Here, we aim to provide an editorial overview on the history of the Hygiene Hypothesis and related topics to offer a framework for the articles collected in the special edition research topic “The Hygiene Hypothesis and its Immunological Implications.”

## A Chronological Overview

The epidemiological basis for the Hygiene Hypothesis became apparent long before the Hygiene Hypothesis was postulated. Two simple observations were made in the 1960s and in the 1970s. First, a Swedish study described differences in the prevalence of asthma and socio-medical conditions between populations living in urban or rural sites (2). A few years later, in a population-based study conducted in Saskatchewan, Canada, showed that allergies were less frequent in native tribes living traditionally in rural sites compared to Caucasian Canadians living in urban habitats (3). Moreover, the authors postulated that frequent bacterial infections in childhood might be responsible for the inverse association with allergic diseases. Strachan's observations made in the late 80s in a British population corroborated these findings and he later named this concept “Hygiene Hypothesis” in 2000. Briefly, Strachan suggested that transfer of early childhood infections between siblings is associated with protection against allergies later in life (4).

The hypothesis was further substantiated and extended by studies that compared asthma and allergy prevalence directly after the “Fall of the Iron Curtain” between Western and Eastern Germany, a decade later (5, 6). Interestingly, these studies triggered a paradigm shift in allergy research. Until then, environmental pollution was broadly regarded as the leading force for allergy development. Environmental data clearly indicated a higher level of pollution by industrial emissions in Eastern Germany compared to the Western part and the study team therefore hypothesized that the prevalence of allergic diseases was higher in children from Eastern Germany. Surprisingly, the researchers found their hypothesis disproved, as children in Western Germany showed a higher prevalence of allergies. Hence, it was postulated that other exposures than pollutants influence the development of atopic diseases. Socio-demographic and –economic factors, as well as household hygiene turned out to be further discriminatory factors between both parts of German population. Improved sanitation and hygiene were positively associated with atopic diseases. Another decade later a follow-up further validated this hypothesis and found life-style differences and the prevalence of atopic diseases began to equilibrate within 10 years after the reunification. In consequence, the Hygiene Hypothesis became the leading postulate to explain underlying relationships and mechanisms for the development of allergic diseases in a societal context (6).

Based on this paradigm shift, Rook published the “Old Friends-Hypothesis” which argues that infectious diseases have a long co-evolutionary history with human development, and appropriate levels of exposure to these microorganisms early in life might protect against immune deviation and allergic diseases. These early-life exposures to potential pathogens might educate the developing immune system from a type-2-dominated *in utero*-milieu toward a more defensive T helper (h)1 response (7).

The next milestone involved findings obtained from the so-called “Alpine farm studies” conducted at the turn of the millennium. Von Mutius and Braun-Fahrländer recognized the unique situation that the Alpine traditional farming environment represents a socio-cultural and ecological niche which significantly differs from the post-modern and urbanized life-style. In a number of epidemiological studies they identified traditional farming characteristics such as consumption of unprocessed farm milk and close contact with farm animals to act allergoprotective and found these parameters to be associated with a higher microbial load. These Alpine farm studies added substantial evidence to Strachan's basic idea and led to a broader view and understanding of the relationship between human health and (early life) exposure to microbes (8, 9).

Further evidence was added by studies conducted in Northern Europe. In the late 1990s studies conducted in Scandinavian und Baltic children described microbial factors to be associated with a lower prevalence of allergic diseases in the Eastern countries (10–12). Next, the Karelia Study, conducted on both sides of the Finnish-Russian border, addressed the impact of the environmental microbial burden on the development of allergic diseases in Finnish and Russian Karelian children that share the same ethnic background but have different life-styles (13). These studies corroborated the Alpine farm studies and point to the microbial environment as a major factor in allergy development.

Furthermore, these studies demonstrate that the diversity and the richness of an immune-stimulating microbial world in human habitats is crucial to establish a competent, tolerogenic and defensive immune system configuration while absence or depletion of those stimuli as found in post-modern environments foster immune deviation and development of allergic diseases (14).

Moreover, two relevant studies [the cross-sectional study “Prevention of Allergy Risk factors for Sensitization In children related to Farming and Anthroposophic Lifestyle (PARSIFAL)” and the multi-center, pregnancy/birth cohort study “Protection against Allergy: Study in Rural Environments (PASTURE)”] support the idea that the “window of opportunity” in which the appropriate education of the immune system starts already in the mother's womb (15–17). The PARSIFAL Study demonstrated that maternal exposure to a farm environment rich in microbial compounds is inversely associated with the development of atopic sensitization and correlated with an upregulation of receptors of the innate immune system in the offspring at school age (15). Further, maternal farm activities during pregnancy were shown to modulate cord blood cytokines and allergen-specific immunoglobulin responses toward a Th1 pattern (16, 17). These findings are in line with the Barker theory (18), postulating that pathological pathways occurring in adolescence and adulthood are paved already in prenatal life.

## The Hygiene Hypothesis and the Bacterial World

Even before high-throughput sequencing techniques were established that allow a deeper view into the microbial world on our body surfaces, Noverr and Hufnagle proclaimed the “Microbiota Hypothesis” by which they claimed the microbiota to be indispensable for developing and maintaining a tolerogenic immune status (19). A similar idea concept was proposed earlier by Holt, Sly and Björkstén (20). The rediscovery of the microbiota and its powerful metabolic and immunologic interplay with the mucosal surfaces of the host underlined and complemented the principals of this basic idea (21, 22). Microbiome research has made significant achievements over the past 15 years; here we can emphasize only a few aspects that might be relevant in the context of the Hygiene Hypothesis and the development of allergies.

### Phylogenetic Impacts

An intriguing concept to better understand the complex symbiotic interplay at organ surfaces was suggested by McFall-Ngai in 2007. In her evolutionary perspective she shed light on findings made in invertebrates which not only lack an endoskeleton but also an adaptive immune system. Thus, invertebrates have to exclusively rely on their innate immune system, which to our current understanding, lacks an immunological memory. Analyses of the intestinal microbiota in such animals have shown—in contrast to vertebrates—a rather low diversity in the community of their microbial residents. Only a handful of strains could be identified as stable colonizers on the gastrointestinal surfaces while most bacteria travel through as transient visitors. Some invertebrates, like insects, separate bacterial colonies from epithelial host cells by a peritrophic matrix composed of chitin and other compounds (24). During the course of evolution, the microbial colonization of epithelia started to get more complex and in turn the host was challenged to develop new strategies to manage these diversifying communities. To permanently recognize a specific bacterium as beneficial or harmful, an adaptive immune response that provides an immunological memory over generations of immune cells was needed. Mutual adaption of both partners, the bacterial community, as well as the complex network of adaptive immune cells, led to a sophisticated metabolic and immunologic interplay with a highly digestive and defensive performance. This symbiosis is based on early education of the host's immune cells by a diverse microbial community to successfully discriminate dangerous pathogens from beneficial symbionts and own healthy cells. Finally McFall-Ngai stated, that complex systems might be prone to failure and allergies and autoimmune disorders might be a consequence of this (23).

### Ontogenetic Impacts

A number of recently published reports substantiated the impact of the early life microbiota on immune maturation [recently reviewed in (25)] and the development of allergic disorders in early infancy [recently reviewed in (26)]. The developmental starting point of the infant gut microbiota is still unknown, but undoubtedly, the process of delivery seems to be a key point in the development of the neonatal microbiota (27). Meconium, the neonate's first intestinal discharge, was shown to contain various bacterial strains indicating that the perinatal gut is colonized by bacteria (28, 29). In a landmark study, Dominguez-Bello et al. reported that the neonatal microbiota differs between vaginally born infants and neonates delivered by Caesarian (C)-section. The authors found a high abundance of *Bacteroides, Bifidobacterium*, and *Lactobacillus spec*. in meconium samples obtained from vaginally delivered newborns, while *Staphylococcus, Streptococcus, Corynebacterium*, and *Propionibacterium spp*. were found predominantly in meconium samples of C-section born neonates (30).

Colonization of the neonate's colon by *Lactobacilli* and *Bifidobacteria* transferred from the maternal vaginal compartment during vaginal passage might provide advantages for the newborn due to the metabolic properties of these bacteria that foster the adaptation to milk-based feeding. These bacteria are capable of metabolizing breast milk-derived lactose and human milk oligosaccharides (HMOS) (31) and were shown to provide immune-modulating short chain fatty acids (SCFAs) (32) and conjugated trans-linoleic acids (tCLAs) (33), which are shown to reduce pro-inflammatory eicosanoid production by regulating the transcription of cyclooxygenase 2 (COX-2) (34) and to induce anti-inflammatory M2-macrophage differentiation (35).

However, how sustainable and decisive are these mode of delivery-associated differences beyond the neonatal age? Chu et al. recently showed that function and composition of the microbiota significantly diversifies in all body sites within the first 6 weeks of life, resembling the corresponding maternal body site microbiota at this time point. Infant's mode of delivery or other prenatal factors seems to have no impact on this development (36). Data from the Copenhagen Prospective Studies on Asthma in Childhood_2010_ (COPSAC_2010_) cohort underlined the importance of the maturation of the microbiota on the further development of the gut microbiome and the risk of asthma later in life. In that study, Stockholm et al. compared the gut microbiome of vaginally and C-section delivered infants from birth to 1 year of life in the context of asthma development at school age. Marked differences between C-section and vaginally delivered infants were observed by 1 week and by 1 month of life, but only minor differences between these groups were found by 1 year of age. An increased risk for school-age asthma was only observed in a subgroup of C-section-born infants that maintained the C-section-associated composition for at least 1 year. The authors conclude that vaginal delivery and/or subsequent maturation of the infant microbiota might support a more robust and stable microbiota in the offspring that is more adaptive to the challenges later in life (37). Further exposure to the maternal microbiota (38), as well as nutritional impacts (e.g., cessation of breastfeeding) (39) within the first month of life, might foster the maturation of the gut microbiome in early infancy.

### Nutritional Impacts

How is the microbiota linked to the rising atopic epidemic observed in the recent decades? A recently published study conducted in indigenous tribes living in the Brazilian Amazonas-Orinoco Basin may help to answer this question (40). In this study the gut microbiome of the semi-nomadic gatherer/hunter people of the Yanomami who maintained a primitive close-to-nature life-style was compared to subjects representing populations that are characterized by a westernized or non-ancestral life-style in rural and urban settings. The Yanomami microbiota was significantly more diverse than those of the westernized counterparts. Moreover, an additional study comparing Venezuelan with Brazilian Yanomami indicated a high level of adaptability to specific environmental conditions of the microbiota in these peoples. While a high taxonomic diversity was found in both sub-tribes, the composition of microbiota was significantly different (41). These findings point to environmental and life-style factors that influence the composition of the microbiota the absence of which may thus foster the loss of taxonomic and metabolic diversity in westernized societies (42).

Diet is one of the most prominent environmental factors that differ between modern and ancient life-styles. While dietary habits in indigenous people such as the Yanomami strongly depend on the sometimes limited food supply due to seasonal cycling, people living in developed societies have access to high in calories food ready at any time and in abundance. Moreover, diet in indigenous cultures is often based on high-fiber products derived from plants that are easy to culture such as plantain, manioc or sweet potatoes, all rich in inulin (43). High-fiber diet and, in particular inulin, is known as an effective enhancer of beneficial bacteria such as *Bifidobacteria* in the colon that stabilize gut homeostasis (44). Translating these findings into a clinical approach, McLoughlin et al. applied soluble inulin to asthmatics in a short-term placebo-controlled-trial and could report an array of beneficial effects in patients orally treated with inulin. In comparison to the placebo group, inulin-treated patients displayed a significantly reduced number of eosinophils in the sputum and, overall, reported a significantly improved asthma control. Inhibition of histone deacetylase 9 (HDAC9) in sputum cells upon a combined application of inulin and a multi-strain probiotic mixture of *Lactobacillus acidophilus, Lactobacillus rhamnosus* GG and *Bifidobacterium animalis* subspecies *lactis* indicated that epigenetic pathways are involved in the mechanisms by which lactic acid bacteria modulate host responses in combination with the prebiotic gavage (45).

A number of recently recognized metabolites released by beneficial symbiotic bacteria convey immunomodulatory effects, mainly in the gut but also on other mucosal surfaces (46).

In particular, SCFAs derived from dietary fibers and released in the lumen of the colon contribute to immune modulation and inhibition of pro-inflammatory cytokines when absorbed by gut epithelial cells (47). By binding to chemoattractant G protein 43 receptor, SCFAs are capable of regulating inflammatory responses (48) as shown for intestinal inflammation (49). Tryptophan, an amino acid produced by an array of beneficial microorganisms, is degraded to indole derivatives which may bind to the aryl hydrocarbon receptor (AHR) and by this regulate the activity of immune cells at the epithelial barrier. That involves AHR-dependent differentiation of regulatory T cells associated with anti-inflammatory IL-10 expression. Further, Th2-cells are inhibited on the transcription factor level in favor of a Th1 response (50).

A number of beneficial bacteria contribute to the orchestration of T cell subsets at the gut epithelial barrier. *Bacteroides sp*. and *Clostridium* clusters IV and XIVa colonizing the gut epithelium are known to stimulate intestinal epithelial cells to release thymic stromal lymphopoietin (TSLP), transforming growth factor (TGF)-ß and interleukin (IL)-25 which in combination may induce tolerogenic effects in dendritic cells (DCs) (51), e.g., by secretion of TGF-ß and retinoic acid. Both factors initiate differentiation of naïve T cells to regulatory T cells upon activation of the nuclear transcription factor forkhead box P (FoxP3) (52). These regulatory mechanisms are challenged by “pathobionts” or other damage factors. In presence of these stressors, overexpansion of Th1, Th2 and Th17 effector cell subsets might result in an inflammatory response in the infected organ or, by migration of these cells, at distant sites. Namely, *Clostridium difficile*, which is associated with wheezing and atopic sensitization, was shown to initially disturb the intestinal balance when acquired early in childhood (53).

Traveling from the gastrointestinal to the respiratory tract the microbiota established in the lung might also play a role in the development of allergic disorders, namely of allergic asthma. Though the gut is known to play a major role in establishing and regulating immune defense mechanisms, the “gut-lung axis” alone might not completely explain the rise of allergic asthma (54). As many studies focused on the lung microbiome, it has become clear that there is a strong relationship between frequently inhaled environmental microbes, microbial colonization of the respiratory tract, and the prevalence of allergic asthma (55). For example, results from the “Multidisciplinary Study to Identify the Genetic and Environmental Causes of Asthma in the European Community (GABRIEL) Advanced Studies (GABRIELA)” study suggested a transfer of built-environment-associated bacteria into the respiratory tract. Indoor dust samples from farm houses and nasal swabs from farm children displayed a higher bacterial diversity than those samples collected in rural non-farm children (56). New evidence was added recently by studies conducted in the Finnish part of the PASTURE-study. Kirjavainen et al. reported that the ecological diversity of the so-called “indoor microbiota” is inversely linked to the prevalence of allergic asthma. Substantiating former farm studies, this report further validated the hypothesis that microbial diversity and composition in the natural environment is linked to a reduced risk of early-onset allergic asthma and that traditional farming is a proxy for this effect (57).

But what are the cellular and molecular mechanisms associated with high microbial diversity? Interestingly, the farm studies consistently showed an inverse association between a highly diverse environmental microbiota and allergic asthma, but this did not account to other allergic manifestations such as hay fever or atopic sensitization. On the other hand, endotoxin exposure protects against atopy but fosters the risk of non-allergic asthma and early onset of wheeze when inhaled in higher concentrations. These findings derived from the farm studies still challenge the Hygiene Hypothesis and might point out that microbial colonization and exposure to microbial compounds have to be considered separately (58). Integration of beneficial environmental bacteria into the microbial community of the respiratory tract leads to a tolerogenic mucosal symbiosis that establishes a local T-cell balanced anti-inflammatory milieu at the epithelium, probably enhanced by a well-balanced gut microbiota. Endotoxins are potent activators of innate TLR-signaling and can attenuate B cell driven sensitization and formation of IgE-antibodies (59). Already in 2003, Vercelli postulated a switch from Th2-driven allergic responses at low endotoxin exposure to a pronounced Th1 response in the lung under high levels of environmental endotoxin. This might explain the elevated prevalence of non-allergic asthma in environments overloaded with endotoxin (60).

## Conclusions

The many aspects and facets of the Hygiene Hypothesis have been supported by concepts and findings coming from a variety of scientific disciplines such as epidemiology, immunology, microbiology and anthropology. Within the last three decades we obtained a multiplicity of new insights into the complexity and plasticity of T cell networks which led us to recognize the complexity and significance of a powerful and well-regulated adaptive immune response in relation to exogenous factors (61). Early developmental findings characterizing pre and postnatal life events highlighted the initial role of the innate immune system as an early warning system that orchestrates, educates and shapes subsequent immune responses (62, 63). Evidence from evolutionary biology and anthropology enabled us to understand how host-environment interactions are refined throughout evolutionary adaption (58, 64). Microbiology added fundamental knowledge about the micro-ecosystem that is established throughout the human body as a unique symbiosis between humans and microbes. And finally, coming back to the introductory statement, epidemiological observations such as those initially made by Strachan and von Mutius about 30 years ago still challenge and refine the hypothesis.

## Author Contributions

All authors listed have made a substantial, direct and intellectual contribution to the work, and approved it for publication.

## Conflict of Interest

The authors declare that the research was conducted in the absence of any commercial or financial relationships that could be construed as a potential conflict of interest.
